# Clinical utility of artificial intelligence models in radiology: a systemic scoping review of diagnostic and endovascular applications

**DOI:** 10.1186/s42155-025-00573-8

**Published:** 2025-10-30

**Authors:** Som P. Singh, Aarya Ramprasad, Mina S. Makary

**Affiliations:** 1 University of Texas Health Sciences Center at Houston, Houston, TX USA; 2 University of Missouri Kansas City School of Medicine, Kansas City, MO USA; 3 Division of Vascular and Interventional Radiology, Department of Radiology, The Ohio State University Wexner Medicine Center, 395 W 12Th Ave, 4Th Floor Faculty Office Tower, Columbus, OH 43210 USA

**Keywords:** Artificial intelligence, Interventional radiology, Deep learning, Personalized medicine, Scoping review

## Abstract

**Background:**

To systematically scope the clinical integration of artificial intelligence (AI) in diagnostic and interventional radiology. This integration encompasses various components of AI forms such as deep learning, convolutional neural networks, natural language processing, and machine learning.

**Methodology:**

A Preferred Reporting Items for Systematic Reviews and Meta-Analysis Extension for Scoping Reviews (PRISMA-ScR) was employed to evaluate current primary and translation literature on the utility of AI in diagnostic and interventional radiology in broad disease categories.

**Results:**

Following the review for inclusion criteria, a total of 23 peer-reviewed research articles were selected for review. Notably, most studies were found to focus on diagnostic and interventional radiology and oncologic diseases, including lung, hepatocellular, colorectal, prostate, pancreatic, breast, and blood cancers.

**Conclusions:**

Radiologists have an advantageous role with the integration of these tools in clinical practice. This may include disease prediction models, catheter navigation, and image reconstruction. Utilization of these AI tools can help improve and further expose of the capabilities of diagnostic and interventional radiology to patients worldwide. From a disease standpoint, this review found most of the clinical literature has implemented AI tools for diagnostic and interventional radiology in oncology, followed by vascular diseases. Careful navigation is necessary to address the current logistical challenges, educational demands, and ethical dilemmas to ensure the safe and effective incorporation of these technologies into clinical radiologic settings.

## Introduction

Artificial intelligence (AI) modalities are emerging tools for healthcare providers to enrich patient care. Within diagnostic and interventional radiology, a growing body of evidence profiles the possible application of AI-powered large language models (i.e., ChatGPT, Copilot) in patient education and communication at the pre-, intra-, and post-procedural stages [1–3]. However, the utility of this AI can spread beyond these stages, including in research, healthcare administration, and communication between medical providers across disciplines [4]. Language models, a subtype of AI, are designed to understand and generate human-like text [2]. These models leverage natural language processing techniques to analyze and interpret textual data, enabling a wide range of applications in healthcare, including medical image analysis, clinical documentation, and decision support. In the context of diagnostic and interventional radiology, AI language models offer unique capabilities that can streamline workflow processes, improve diagnostic accuracy, and facilitate data-driven decision-making [1, 5].

Historically, early research and investigation in the mid-twentieth century on natural language processing provided a foundational catalyst for future language modeling [6, 7]. Specifically, pioneering work by researchers such as Alan Turing laid the theoretical foundations for understanding language structure and syntax and the eventual descriptors of AI [5, 7]. However, AI spans beyond natural language processing. In addition, its core pillars include but are not limited to cognitive computing, computer vision, deep learning, machine learning, neural networks, robotics, and speech processing [8–12].

For radiologists, there are numerous potential advantages to using artificial intelligence; however, the literature is still in its early stages. The use of procedures by diagnostic and interventional radiologists is growing to a demand where added modalities of assistances can help improve workflow in this clinical discipline. Though many AI tools are primarily diagnostic in nature, this review aims to highlight their ancillary relevance for interventional radiologists as well as discuss Ais introductions into the procedural IR world. This review aims to provide a comprehensive overview of the clinical utility of AI in radiology, encompassing its current applications, challenges, and prospects.

## Methodology

Given that this was a narrative review, the study followed the Preferred Reporting Items for Systematic Reviews and Meta-Analysis Extension for Scoping Reviews (PRISMA-ScR) [13]. The search strategy employed in this narrative review evaluated the current primary and translation literature on the utility of AI in radiology and related subsets. Moreover, particular attention was placed on examining the attributable effects of AI in broad disease categories. Following this structured approach, the relevant literature was screened from PubMed (1946 to present), Cochrane Library (all available dates), and Epistemonikos (all available dates). The search query used the following terms in string combinations: “artificial intelligence” AND “interventional radiology” using Title/Abstract in PubMed, Title/Abstract in Epistemonikos, and Title/Abstract/Keyword in Cochrane Library. Articles were reviewed for title and abstract, followed by full-text review if deemed appropriate by the authors of this study. Inclusion criteria for the selection of the articles were determined as follows: 1) Involved the utilization of radiologists in clinical care, 2) a clear description of the artificial intelligence methodology used, 3) a clear description of clinical study outcomes, and 4) article availability in full-text, English language. The exclusion criteria were also as follows: 1) the study did not provide a clear description of the artificial intelligence models utilized, 2) the article was not a full-text peer-reviewed journal article (i.e., conference proceedings), 3) duplicate article, and 4) non-English language article.

All articles collected from the literature were subsequently downloaded into Endnote X9® with subsequent study selection using Covidence® [14]. A narrative approach to synthesize the extracted data from the included studies was utilized as the study was categorized by disease management whenever applicable [15]. Specifically, each article was reviewed to determine the area of diagnostic and interventional radiology targeted in this study (i.e., diseases, interventions) and were synthesized by disease category. This screening and review process was carried out by two reviews (S.P.S. and A.R.). If any disagreement was made between the two reviewers, the senior author (M.S.M) was consulted to aid in confirming a decision. Moreover, this study did not require institutional review board approval, considering that there were no human or animal subjects participating in the study. Given the nature of this study design, institutional review board approval was not required.

## Results

**Fig. 1:**
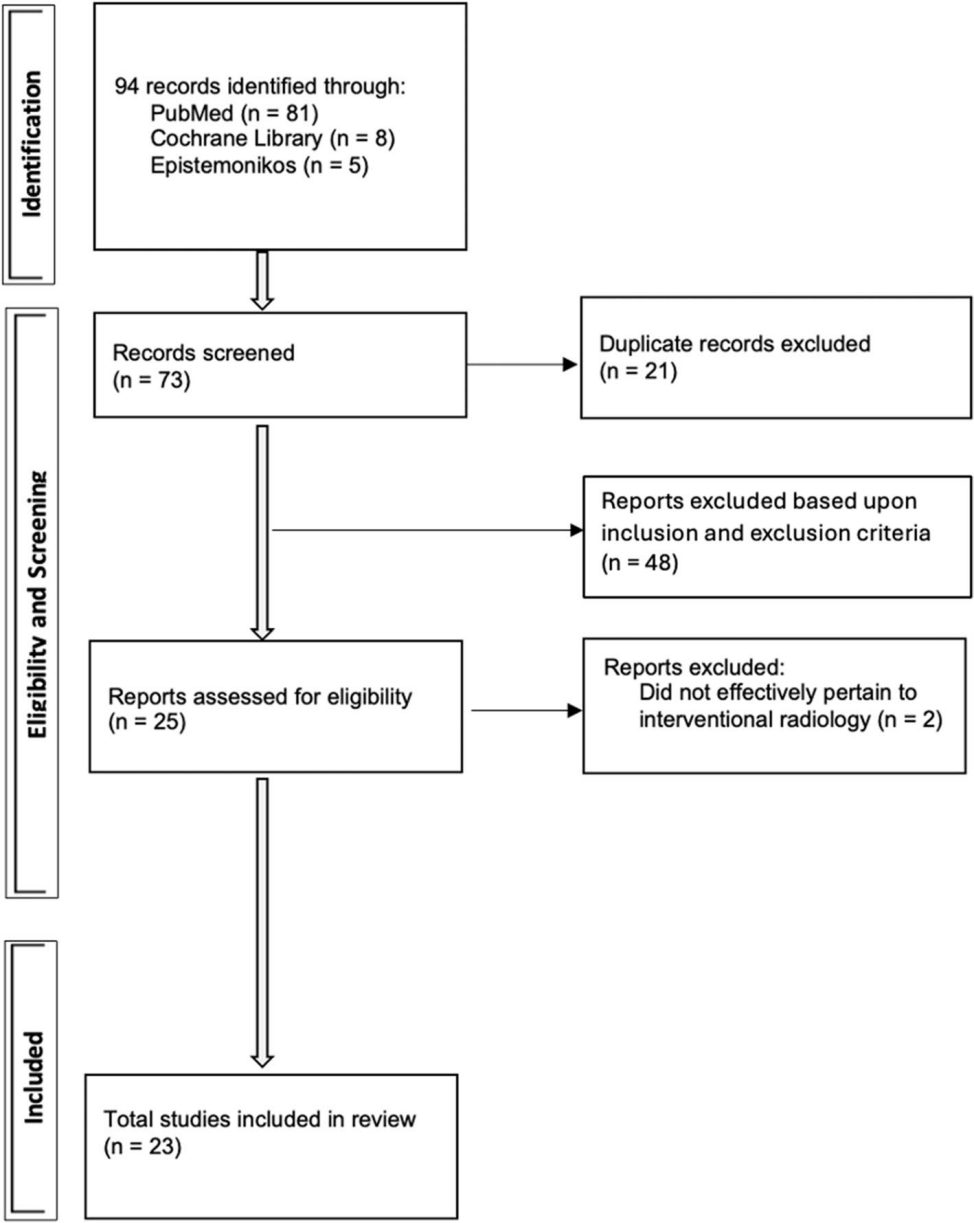
Study selection process of the scoping review. Identification led to 94 records being retrieved for eligibility and screening. A total of 23 studies were included in review to address the primary aim of this study

The employed search strategy for this scoping review initially identified 94 studies, with publication years ranging from 2013 to 2024, with initial strings applied (PubMed = 8; Epistemonikos = 5; Cochrane Library = 8). From this, 73 articles were retained after screening for full-text review based on initial exclusion criteria. Following the review for inclusion criteria, a total of 23 peer-reviewed research articles were selected for narrative review, as demonstrated in Fig. 1. The research articles were found to have been published between 2019 and 2024, with the most being published in the year 2022 (*n* = 6). Among these studies, the dataset of samples or patients was most often found to range between 100 to 999 samples/patients (*n* = 11), and only two research articles were comprised of over 5000 samples/patients.

A majority of the research studies investigated artificial intelligence in the management of oncologic diseases (*n* = 19). Within this subset, colorectal, lung, pancreatic, and prostate cancer comprised the most at 3 each. Additional disease processes that were found to have been investigated included vascular disease (*n* = 1) and adult polycystic kidney disease (*n* = 1), osteoporosis (*n* = 1). The following discussion highlights different interventions that exist within different disease subtypes (Table 1).

**Table 1:** Summary of studies from search process. Study characteristics included publication year, sample size of study, and disease discipline

Study Characteristics	Included Studies
Publication Year:	
2019	1
2020	4
2021	3
2022	6
2023	4
2024	5
Sample Size:	
< 100	2
101–999	11
1000–5000	8
> 5000	2
Discipline:	
Colorectal Cancer	3
Lung Cancer	3
Prostate Cancer	3
Pancreatic Cancer	3
Breast Cancer	2
Renal Cell Cancer	1
Hematologic Diseases	1
Hepatocellular Cancer	1
Glioblastoma	1
Vascular Diseases	2
Polycystic Kidney Disease	2
Bone Mineral Diseases	1

### Segmentation

In prostate cancer, biopsies and image-guided treatments often rely on ultrasound combined with CT/MRI for guidance. The accuracy of image fusion depends heavily on precise segmentation of the prostate in ultrasound images. AI has shown remarkable capability in assisting image reconstruction and achieving accurate prostate gland segmentation in transrectal ultrasound images, despite challenges such as low signal-to-noise ratio and the presence of artifacts [16]. In adult polycystic kidney disease (APCKD), total kidney volume (TKV) is a significant biomarker for evaluating disease severity. Manual tracing of organ boundaries is both time-consuming and variable among readers [17, 18]. A study by Woznicki et al. presented a deep learning model that automates segmentation of both kidneys and liver, demonstrating a precise and reliable method for estimating TKV [18]. Additionally, in hematologic disease diagnostics, a study by Fervers et al. used AI-driven segmentation to identify non-fatty portions of bone marrow from spinal imaging. This segmentation correlated with a higher degree of marrow infiltration, even after adjusting for bone mineral density [19]. In regard to endovascular procedures, specifically embolic procedures, real-time intraoperative guidance during the repair of intracranial aneurysms is provided by the Aneurysm Occlusion Assistant (AnOA) software. This tool integrates AI-driven lesion segmentation to segment cranial vasculature and aneurysms automatically, with a dice index of ~ 0.78 and occlusion prediction accuracy of 0.84, enabling clinicians to make swift therapeutic adjustments [20]. Furthermore, AI-driven vessel segmentation for endovascular aneurysm repair (EVAR) procedures has been explored, where deep learning algorithms help identify optimal landing zones for stent graft deployment, improving procedural accuracy and avoiding suboptimal zones [21]. In addition to that, AI has also been incorporated into real-time catheter guidance systems, like the MR-based system that Elgort et al. developed, which realigns image parameters in real-time based on catheter movement, significantly enhancing image quality and procedural safety during procedures like embolization. The adaptive system has also been shown to localize a moving catheter accurately, and maintain images to offer precise guidance [22].

### Prediction

AI models have demonstrated strong performance in prediction tasks across various cancers. In lung cancer, the Lung Cancer Prediction Convolutional Neural Network (LCP-CNN) uses deep learning to generate malignancy scores for nodules on CT, ruling out malignancy in about 20% of patients with nodules ranging from 5 to 15 mm [23]. For hepatocellular carcinoma (HCC), convolutional neural networks trained on CT data have outperformed traditional radiomics in forecasting overall survival, offering important prognostic insights irrespective of tumor stage or treatment [23]. In colorectal cancer, neural networks have been used to predict prognostic biomarkers from histopathology specimens, enabling risk stratification to tailor patient management [24, 25]. These AI-driven models also assessed the prognostic value of risk groups after adjusting for known variables [26]. AI models have also been applied to locally advanced gastric cancer (LAGC). A deep learning radiomic nomogram developed by Dong et al. outperformed standard clinical N stages, tumor size, and clinical models in predicting overall survival [27]. In pancreatic cancer, neural networking has evaluated sarcopenia to assist in prognosis [28, 29]. Wang et al. also introduced a tool for individualized prognostic evaluation [28]. For vascular disease, deep learning has been used to predict major adverse limb events and 5-year all-cause mortality from posterior tibial arterial Doppler waveforms [30]. AI technologies have shown promise in the management of peripheral artery disease (PAD) as well. For example, AI models derived from Doppler waveforms can accurately identify PAD, enabling early diagnosis and detection. Lareyre et al. also demonstrate how AI, particularly machine learning and computer vision, facilitates PAD diagnosis and prognosis through automated artery lesion detection and optimization of screening procedures [31]. Furthermore, AI-based models, such as a Markov-based model developed by Brothers et al. (2023), were employed in predicting the optimal therapies for PAD patients in a bid to maximize patient quality of life (cQoL) after interventions. The model demonstrated that AI can predict treatment outcomes accurately, maximizing patient care plans via the prediction of probable clinical outcomes with strong accuracy [32]. Regarding vascular access pathway planning and selecting embolization material, during the complex repair of aortic aneurysms, AI-assisted tools, including Surgical Augmented Intelligence, have been observed to decrease exposure to radiation, fluoroscopy duration, and the use of contrast, in addition to enhancing procedural planning. This technology allows decision-making within intricate interventions by combining real-time fluoroscopic images with patient-specific information to enhance operator efficiency [33]. Recent studies have also identified the potential of AI in facilitating real-time embolization during the treatment of intracranial aneurysms. One AI system was able to exhibit high accuracy in detecting liquid embolic agents from biplane fluoroscopy images, notifying physicians when the agent reached goal targets. This helped prevent complications that could arise from accidental embolization of vessels. This system exhibited high notification accuracy (100%) and recall (92%), emphasizing the potential of AI in ensuring clinical safety and efficacy in real-time vascular interventions [22].

### Radiomics

In breast cancer, AI applied to MRI radiomics has proven useful for preoperative assessment of axillary lymph nodes [34]. Post-intervention, deep learning models have been trained to evaluate recurrence risk following surgery [35]. Moreover, AI has facilitated the exploration of long non-coding RNAs as potential epigenetic markers of recurrence [36]. In LAGC, radiomics combined with deep learning has enabled the creation of nomograms that surpass traditional clinical indicators in survival prediction [37, 38]. In HCC, both traditional radiomics and deep learning techniques have been explored for survival forecasting using CT imaging, with deep learning showing superior predictive power [30]. For vascular diseases such as PAD, AI algo ithms have been employed to analyze radiomics from CT and Doppler waveforms and have been found to predict unfavorable clinical events and enhance screening programs for PAD [31].

### Computer-Aided Detection (CAD)

In lung cancer, delayed diagnosis due to subtle symptoms is common and leads to poorer prognosis [37, 39]. Early detection is crucial, and AI-based CAD systems such as EIRL Chest X-ray Lung Nodule (LPIXEL Inc.) have demonstrated value in detecting nodules on radiographs [19]. These systems assist in localizing biopsy targets for pulmonologists and interventional radiologists. Further more, Barash et al. trained a convolutional neural network to detect active bleeding from digital subtraction angiography images on patients undergoing celiac artery and mesenteric procedures, at 85.4% sensitivity and 81.2% specificity, spotlighting the coming importance of AI in acute vascular imaging diagnoses [38]. While CAD tools support radiologists in timely communication with other specialties, they may offer even greater benefit to general physicians by accelerating initial clinical decision-making.

### Dose optimization

AI has been used to enhance the diagnostic quality of PET images from low-dose scans, addressing a critical challenge in reducing patient exposure while maintaining image quality [24]. However, AI has not yet achieved the ability to reduce the radiotracer dose to 1% of the clinical standard, revealing limitations in its current capabilities [24–26]. In intricate endovascular procedures, surgical augmented intelligence technology has also been observed to decrease significantly radiation exposure (1955 mGy vs. 3755 mGy) and contrast volume use (123 mL vs. 199 mL) with improved intraoperative safety [33].

### 3D reconstruction

In cerebrovascular disease management, accurate localization of intracranial aneurysms is critical. A study by Zhao et al. developed a 3D digital subtraction angiography reconstruction (SDR) network capable of reconstructing 3D-DSA images from fewer 2D projection views, optimizing workflow and reducing exposure [40].

### Multimodal imaging alternatives

For prostate cancer, AI has been explored as a cost-effective alternative to PSMA PET/CT by predicting lymph node status using contrast-enhanced CT alone. Deep learning models demonstrated encouraging outcomes in this regard [41, 42].

### Optimization in biopsy and interventions

AI supports diagnostic bone marrow biopsies in hematologic malignancies such as leukemias and lymphomas [43–46]. In APCKD and renal failure workups, AI assists interventional radiologists by streamlining volume assessments and reducing reader variability [47–49]. In breast cancer, AI aids interventional and diagnostic radiologists in biopsy planning and staging.

In summary, AI applications span a range of techniques including segmentation, prediction, radiomics, CAD, and dose optimization, each contributing to improved diagnostics and workflow in diverse clinical settings.

## Discussion

The integration of AI into healthcare is rapidly transforming the landscape of patient care, particularly within radiology. This integration has occurred in numerous forms, including but not limited to deep learning, convoluted neural networks, natural language processing, machine learning, etc. Through a structured search strategy, a total of 23 peer-reviewed research articles were identified, spanning various disease domains and procedural stages [16, 18, 19, 23, 24, 26–30, 35, 41, 42, 50–58]. Notably, the majority of studies focused on oncologic diseases, including lung, hepatocellular, colorectal, prostate, pancreatic, breast, and blood cancers, reflecting the breadth of AI's impact on oncologic care.

The current state of AI is able to span numerous oncologic diseases in a similar fashion. Moreover, the ability to span numerous oncologic diseases was similarly performed through frequent utilization of deep learning. This broad spectrum of AI applications is useful to foster collaboration between clinical specialties, such as radiologists, primary care providers, and oncologists. Such collaboration can create more impactful, patient-centered care pathways and accelerate the deployment of AI into real-world clinical workflows. The improved collaboration could educate non-radiologic disciplines on the recommendations provided by the expertise of radiologists in a more convenient nature. For example, a patient undergoing workup for the clinical suspicion of certain cancers may be referred by a primary care physicians or oncologist for a biopsy to further advance care. However, the availability of an immediate biopsy can be an obstacle for numerous hospital systems worldwide [36, 59, 60]. This may be due to the shortage of radiologists and other specialists trained to obtain a biopsy, the ready-availability of resources to undergo image guidance (i.e., anesthesia, fluoroscopy suite availability), or comorbidity-associated complication risks of undergoing biopsy. To further facilitate this collaboration, interaction between AI developers, healthcare practitioners, and policymakers must be encouraged. A well-trained model trained on deep learning may possibly be able to serve as an additional diagnostic avenue for clinicians to stratify disease prediction and prognosis more immediately while waiting for the patient to undergo a successful biopsy. The creation of shared training programs and standard AI curricula will also help seamlessly integrate into healthcare systems and ensure a quicker process of incorporating AI into patient care...

While oncologic disease literature for AI is well established, there was less literature available in the past for AI in interventional radiology for non-oncologic primary conditions—vascular procedures, specifically. There has been, nonetheless, demonstrated promise of novel deep-learning models for vascular disease, as seen in the results of this study. Critical localization of intracranial aneurysms on imaging can allow for timely workflow and activation of the fluoroscopy suite if emergent intervention is required. Moreover, the domain of literature on AI in coronary artery disease demonstrates similar utilization, such as image reconstruction and predictive risk models [43–46, 61]. Further bibliometric analysis better addresses the status of AI in vascular diseases.

Beyond the scope of this search strategy, there are numerous areas of discussion on where AI can serve an innovative role within radiology. One such area is in pulmonary disease, where AI has the potential to screen all CT scans performed in a hospital and alert radiologists and clinicians to cases with urgent findings, such as acute pulmonary embolism. Rothenberg et al. described an innovative AI-driven triage product for the detection of pulmonary embolisms on CT [62]. The study demonstrated that radiologists assisted by this AI system were able to detect PE more quickly than without it. The more rapid detection could lead to an earlier notification of the pulmonary embolism response team and enable timely mechanical thrombectomy [63]. However, diagnostic accuracy and miss rate did not show a significant difference with this tool. Moreover, AI algorithms have been used to identify candidates for endovascular interventions for pulmonary embolism with high sensitivity but poor response team activation [64]; for hospitals that may not have response team availability, implementing a refined algorithm may still provide an advantageous avenue for clinicians to gather data to safely transfer patients for higher levels of care at other hospitals more efficiently. AI may also be utilized to detect deep venous thrombosis well before a pulmonary embolism, as deep learning has shown effective utilization in detecting inferior vena cava filters on CT [40, 65, 66].

The intra-procedural utility of AI may improve catheter guidance as well. For example, machine learning has been utilized to optimize intravascular ultrasound to determine plaque burden and luminal area in cardiac catheterization [47, 67]. Similarly, it may also serve as another tool for optimal catheter tracking, as a study by Annabestani et al. utilized AI to predict the roll angle of an intracardiac echocardiography catheter using values extracted from fluoroscopy images [48]. The body of literature on catheter guidance continues to grow. It may still be in its earliest stage, but promising possibilities may also include autonomous navigation of the procedure. These advancements can assist proceduralists in developing technical skills and open avenues for broadening the capability of endovascular interventions. For both radiologists and patients, a growing body of evidence also explores utilizing AI to reduce radiation exposure during the intervention [1, 49]. Specifically, Bang et al. implemented an AI-powered fluoroscopy system, notably decreasing the scatter effect and patient radiation exposure [49].

The current state of AI is proliferating throughout patient care for radiologists. Still, there is also a growing body of literature that outlines the possible formal integration of AI skill development in medical training [68]. These discussions also include training that is applied to medical students and surgical residents [69–71]. To increase the pace of AI adoption, training programs should be expanded to include specialized curricula in clinical practice so that healthcare providers are able to use AI tools. Additionally, incorporating AI training in medical education for all specialties will enable quicker adoption [72]. This is shown in the results of this study which showcase that this curriculum had successfully improved trainee perception of their knowledge and skills related to AI in radiology. This program can be an early framework for further customization to fit specific needs for integrated interventional radiology graduate medical education [73, 74]. Given the relatively minor cohort of trainees, it is especially imperative to integrate AI training for interventional radiology training so that they can collaborate with other medical specialties that are much larger in cohorts. Likewise, radiologists must be able to match their technical understanding of AI with the ethical implications of this technology as well, and therefore, there needs to be a focus on implementing an internally validated model to clinical practice, although there is currently a paucity of literature that showcases models of this nature.

In addition, artificial intelligence needs to be trained in such a way that it protects patient data from unauthorized access and breaches when in line with data and patient information protection policies. This degree of protection needs to go beyond the ability of a clinician to exert supervision over the helpfulness of artificial intelligence. Likewise, radiologists must also be trained to ensure that patients are fully informed about how their data will be used and obtain explicit consent. This could lead to the inclusion of artificial intelligence as a formal consent in some nature at the hospital system level.

Moreover, a key driving force behind the implementation of AI is its efficacy. In spite of the potential of AI to enhance efficiency, its translation into practice has been slower than anticipated. To move AI adoption forward, further direct comparisons between AI-based systems and conventional approaches on clinical outcomes are required. Large-scale clinical trials must establish the effectiveness of AI in enhancing patient outcomes to prove that AI tools are not only reliable but also safe to apply widely clinically. Radiologists should collaborate to ensure these tools are optimized for patient care as well as to avoid missed diagnoses or errors during procedures.

The nature of this scoping review provided a straightforward methodology to appropriately evaluate the literature, which tangibly investigates AI in clinical practice applicable to radiologists. This scoping review was able to profile where the literature currently stands within the disciplines of radiology. However, this search strategy is not without its weaknesses. This strategy may not have been able to evaluate literature that does not consistently apply to all of the domains of interventional radiology (i.e., vascular diseases). Therefore, there may be a paucity of proper understanding of where the current body of literature truly stands. Moreover, the search methodology could be further modified to include additional search terms such as “deep learning” or other terms that involve AI to possibly change the results of this scoping review. However, a key observation made by this study's authors was that the term “artificial intelligence” was often generalized to discuss various subtypes within artificial narrow intelligence and deep intelligence. This may suggest that clinicians-researchers are less aware of the sub-type and modalities of AI, and the authors hypothesize that the use of more specific terminology in the field of AI will be implemented in the clinical literature over time. Likewise, future scoping reviews may be performed to evaluate AI from the standpoint of all interventional disciplines. Additionally, many promising avenues exist to implement this technology in radiology, so there must be an increased research focus on profiling novel tools and patient outcomes.

While AI has promising potential, the challenges to implementation in clinical practice will be difficult to overcome. The heavy educational burden to providers, along with the resource usage needed for technology implementation, training, and cost, can impede the clinical uptake of AI. Institutionally motivated resistance, data security concerns, and varied rules and regulations also impede progress [1, 75, 76]. Ensuring uniform AI training and interdisciplinary collaboration can help bridge the gap between research and clinical application [1, 12]. For example, the widespread implementation of electronic medical records has affected patient outcomes during the early stages of implementation [77–79].

## Conclusion

In its current state, AI has already served as an impactful tool for diagnostic and interventional radiologists, and there is a continuously growing body of evidence to advance the understanding of this tool further. Moreover, deep learning-based training has shown a significant impact on multiple oncologic diseases, including lung, hepatocellular, colorectal, prostate, pancreatic, breast, and blood cancers. However, the utility of AI extends beyond oncology, with promising applications in vascular diseases, such as peripheral arterial disease and cerebrovascular disease. Despite the promising advancements, implementing this tool in radiology has inherent limitations and challenges. Logistical barriers, educational burdens, and ethical considerations must be carefully navigated to ensure AI technologies' safe and effective integration into clinical practice. As a result, researchers, AI engineers, and radiologists must continue a high degree of collaboration to use this tool to address complex clinical challenges and improve patient outcomes.

## Data Availability

The datasets used and/or analyzed during the current study are available from the corresponding author upon reasonable request.
